# Coumarins from the Herb *Cnidium monnieri* and Chemically Modified Derivatives as Antifoulants against *Balanus albicostatus* and *Bugula neritina* Larvae

**DOI:** 10.3390/ijms14011197

**Published:** 2013-01-09

**Authors:** Zhan-Chang Wang, Dan-Qing Feng, Cai-Huan Ke

**Affiliations:** 1 State Key Laboratory of Marine Environmental Science, Xiamen University, Xiamen 361005, China; E-Mail: wzc_ttt@sina.com (Z.-C.W.); 2 Department of Marine Technology and Ocean Engineering, College of Oceanography and Environmental Science, Xiamen University, Xiamen 361005, China; E-Mail: dqfeng@xmu.edu.cn (D.-Q.F.)

**Keywords:** antifouling activity, *Cnidium monnieri*, coumarins, osthole, structure-activity relationship

## Abstract

In the search for new environmental friendly antifouling (AF) agents, four coumarins were isolated from the herbal plant *Cnidium monnieri*, known as osthole (**1**), imperatorin (**2**), isopimpinellin (**3**) and auraptenol (**4**). Furthermore, five coumarin derivatives, namely 8-epoxypentylcoumarin (**5**), meranzin hydrate (**6**), 2′-deoxymetranzin hydrate (**7**), 8-methylbutenalcoumarin (**8**), and micromarin-F (**9**) were synthesized from osthole. Compounds **1**, **2**, **4**, **7** showed high inhibitory activities against larval settlement of *Balanus albicostatus* with EC_50_ values of 4.64, 3.39, 3.38, 4.67 μg mL^−1^. Compound **8** could significantly inhibit larval settlement of *Bugula neritina* with an EC_50_ value of 3.87 μg mL^−1^. The impact of functional groups on anti-larval settlement activities suggested that the groups on C-5′ and C-2′/C-3′ of isoamylene chian could affect the AF activities.

## 1. Introduction

Marine biofouling on man-made structures causes serious technical and economic problems by threatening mariculture facilities, shipping facilities, vessels, and seawater pipelines [1–6]. Although the metal-based biocides such as tributyltin and cuprous oxide can effectively control biofouling, they also affect non-target organisms and are difficult to degrade [7,8]. Thus, the International Maritime Organization (IMO Resolution A., 895 21, 25/11/1999) established the ban of antifouling coatings containing tributyltin (TBT). Furthermore, the discharge of copper from antifouling paints is currently under scrutiny in many countries, because the cuprous oxide is associated with heavy metal pollution and high environmental risk. Obviously, there is a high demand for new antifouling (AF) agents that are not only effective but also environmentally friendly. Since the natural products already exist in the environment and are commonly biodegradable, it is suggested that these natural products with AF activity are promising and are environmentally friendly alternatives for classical AF agents [3,9–11].

So far, most of the AF natural products have been obtained from marine organisms [9,11–19], but some natural products, which were found in herbal plants, have also proved to be AF agents [20,21]. Compared with marine organisms, many species of herbal plants are easily obtained on an industrial scale because of the rapid development of the herbal industry [22]. *Cnidium monnieri*, one of the most popular traditional herbs, has a wide distribution in China. Its fruits have been used for treatment of impotence, renal disease, dermatosis, and colpitis [23]. The lipophilic extract of *C. monnieri* fruits can significantly inhibit the settlement of cyprids [20].

The barnacle *Balanus albicostatus* and bryozoan *Bugula neritina* were chosen for screening AF agents as model organisms. The barnacle *B. albicostatus* is one of the dominant fouling species in East Asian waters and is a great threat to mariculture facilities, shipping facilities, vessels, and seawater pipelines [24–26]. The marine bryozoan *B. neritina* is an important fouling organism in tropical and temperate waters and attached to seawater cages, buoys and dock pilings at certain times of the year [27–29]. Both *B. albicostatus* and *B. neritina* are excellent species for anti-fouling bioassays because their adults can be easily collected and the larvae can be conveniently maintained in controlled laboratory conditions [20,30].

In this study, four coumarins including osthole have been isolated from the fruits of *C. monnieri*, and have been tested for AF activities against *B. albicostatus* and *B. neritina*. Furthermore, five other coumarins were synthesized from osthole and tested for AF properties. These five synthesic coumarins occuring only in trace amounts in nature were difficult to purify from the plant, but were easy to obtain through a simple synthesis processes. Among these compounds, osthole was the main chemical composition of *C. monnieri* fruits [31]. In China, osthole has been isolated from the widely cultivated herbal plant *C. monnieri* and commercialized as medicine or pesticide [32]. Consequently, osthole is in abundant supply at low price on the market, which makes it possible for large-scale antifouling testing. Also, because these coumarins share a similar molecular skeleton, the relationship between the functional groups and AF activity are discussed.

## 2. Results and Discussion

### 2.1. Isolation, Synthesis and Identification of These Compounds

Four natural products were purified from the fruits of *C. monnieri*, and five other compounds, which occur in trace amounts in nature, were obtained by chemical synthesis. General chemical structure characteristics of these compounds are described in the supplementary materials. The spectrum data were consistent with those of references, and nine compounds were identified as osthole (**1**) [33], imperatorin (**2**) [34,35], isopimpinellin (**3**) [36], auraptenol (**4**) [37], 8-epoxypentylcoumarin (**5**) [38], meranzin hydrate (**6**) [36], 2′-deoxymetranzin hydrate (**7**) [36], 8-methylbutenalcoumarin (**8**) [39], and micromarin-F (**9**) [39,40].

### 2.2. AF Activity of Compounds

The EC_50_ and LC_50_ values of Compounds **1**–**9** against *B. albicostatus* and *B. neritina* are summarized in Table 1. The detailed rates of settlement and mortality against *B. albicostatus* and *B. neritina* for Compounds **1**–**9** are shown in the supplementary materials. The standard requirement established by the US Navy program as a potency criterion for natural antifoulants was that of being active at less than 25 μg mL^−1^ in static bioassays [9]. All compounds except meranzinhydrate (**6**) had EC_50_ values lower than 25 μg mL^−1^ and showed inhibitory activities against barnacle settlement. Among these, Compounds **1**, **2**, **4**, and **7** showed high inhibitory activities against barnacle settlement with EC_50_ values <5 μg mL^−1^. The calculated therapeutic ratio (LC_50_/EC_50_) of greater than one was considered for potential use in environmentally compatible AF coatings [41]. The AF compounds with LC_50_/EC_50_ ratio higher than 15.0 were considered as non-toxic AF agents, and the compounds with a LC_50_/EC_50_ ratio lower than 5.0 were considered as toxic AF agents [19,21]. The recent opinion states that the degradable compounds with a low LC_50_/EC_50_ ratio may still be considered when selecting candidate compounds [19]. The LC_50_/EC_50_ ratio of Compounds **1**, **2**, **4**, **5**, **7** and **8** was higher than 5.0, indicating that these compounds are low-toxicity AF agents against the settlement of *B. albicostatus* larvae. All compounds except for **2**, **3** and **5** showed inhibitory activities against bryozoan *B. neritina* settlement with EC_50_ values <25 μg mL^−1^, and the EC_50_ value of Compound **8** was lower than 5 μg mL^−1^. All compounds showed no significant mortality effect on *B. neritina* at a concentration of 50 μg mL^−1^.

In previous research, we reported that the crude extracts of six common Chinese herbs showed AF activities against the cyprids of *B. albicostatus,* and we also identified two AF compounds from *Sophora flavescens* [20]. In this study, we have identified four AF compounds (osthole, imperatorin, isopimpinellin, auraptenol) from the fruits of another Chinese herbal plant *Cnidium monnieri*. The results further demonstrate the value of herbal plants as a source of AF agents. Osthole (**1**) showed significant inhibitory activities against both *B. albicostatus* and *B. neritina*. Furthermore, some osthole derivatives (**5**, **7**, **8** and **9**) also showed established AF activity. It was suggested that the compound osthole should be considered as a potential lead compound for the design of new AF agents.

### 2.3. Impact of the Functional Groups on Antilarval Settlement Activities

**Figure 1:**
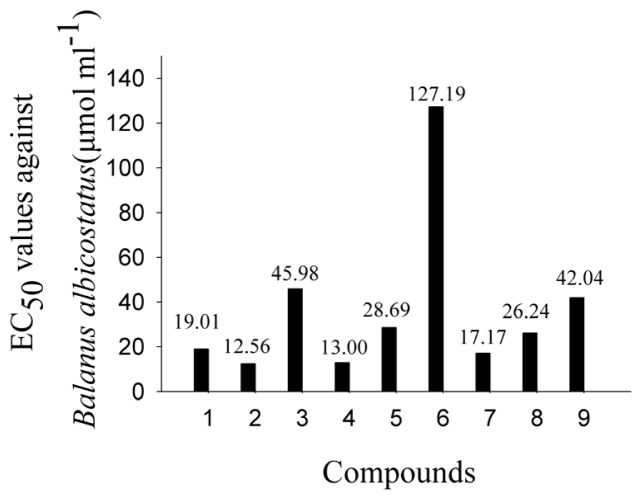
EC_50_ values of Compounds **1**–**9** against larval settlement of *B. albicostatus*.

**Figure 2:**
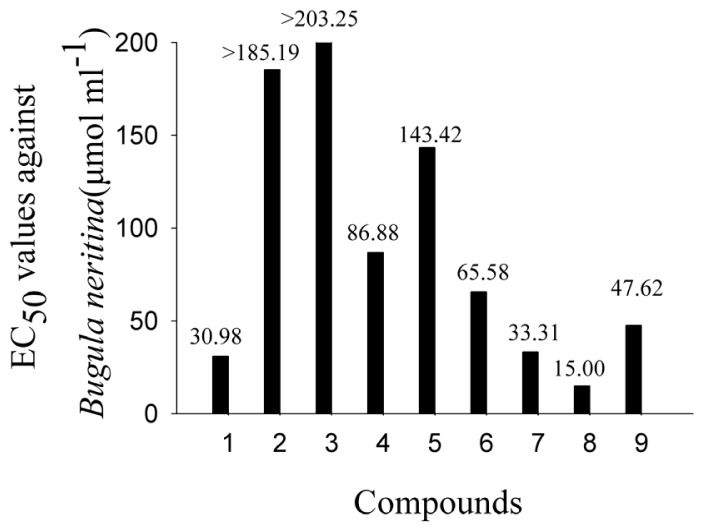
EC_50_ values of Compounds **1**–**9** against larval settlement of *B. neritina*.

Because the nine compounds have a basic coumarin skeleton (benzo-α-pyrone ring) with different functional groups, the impact of functional groups on anti-larval settlement activities could be estimated to obtain preliminary information about the structure-activity relationship (SAR). In order to discuss the impact of functional groups, the concentration unit of bioassay results was converted to micromole per milliliter as shown in Figures 1 and 2.

**Figure 3:**
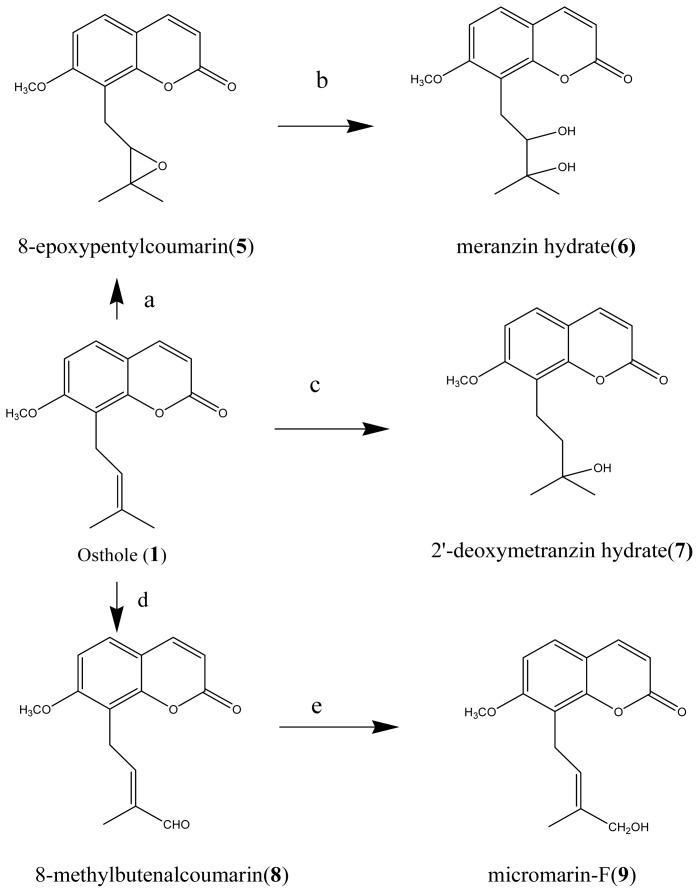
The scheme of the synthetic procedure and the chemical structures of Compounds **5**-**9**. Reagents: (**a**) *m*-CPBA, CH_2_Cl_2_, 0 °C; (**b**) H_2_SO_4_, THF-H_2_O, room temp; (**c**) Hg(AcO)_2_, NaOH, THF-H_2_O, room temp; (**d**) SeO_2_, DMSO-EtOH, reflux; (**e**) sodium borohydride, ethanol.

Compounds **1**, **8** and **9** were the first subgroup to be compared. They have the same coumarin structures, except that the C-5′ groups were 5′-CH_3_ (**1**), 5′-CHO (**8**), 5′-CH_2_OH (**9**), respectively (as shown in Figure 3). The EC_50_ values against *B. albicostatus* were 5′-CH_3_ (19.01 μmol mL^−1^) < 5′-CHO (26.24 μmol mL^−1^) < 5′-CH_2_OH (42.04 μmol mL^−1^), and the EC_50_ values against *B. neritina* were 5′-CHO (15.00 μmol mL^−1^) < 5′-CH_3_ (30.98 μmol mL^−1^) < 5′-CH_2_OH (47.62 μmol mL^−1^). It was suggested that C-5′ groups could affect the activities.

Compounds **1**, **5**, **6** and **7** have the same structures, except that the groups between C-2′ and C-3′ have a double bond (**1**), expoy (**5**), dihydroxy (**6**), hydroxy (**7**), respectively (as shown in Figure 2). The EC_50_ values against *B. albicostatus* were hydroxy (17.17 μmol mL^−1^) < double bond (19.01 μmol mL^−1^) < expoy (28.69 μmol mL^−1^) < dihydroxy (127.19 μmol mL^−1^), and The EC_50_ values against *B. neritina* were double bond (30.98 μmol mL^−1^) < hydroxy (33.31 μmol mL^−1^) < dihydroxy (65.58 μmol mL^−1^) < expoy (143.42 μmol mL^−1^). It was suggested that groups between C-2′ and C-3′ could affect the activities too.

**Figure 4:**
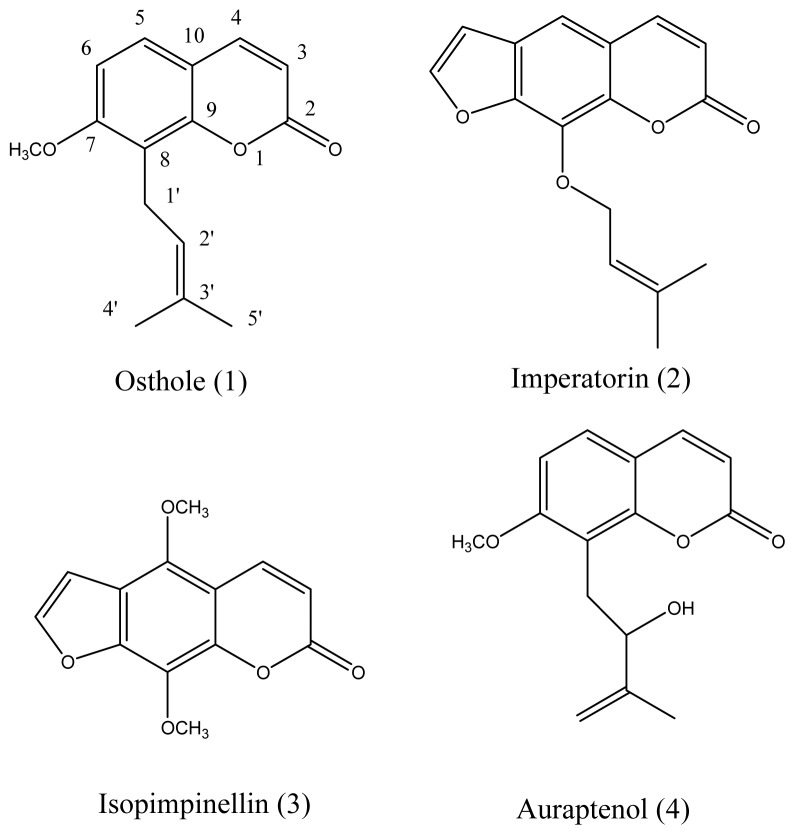
The chemical structures of Compounds **1**–**4**.

The linear furanocoumarin **2** has the same isoamylene chain as simple Coumarin **1** (as shown in Figure 4). They have close EC_50_ values against *B. albicostatus* (Compound **1**, 19.01 μmol mL^−1^ and Compound **2**, 12.56 μmol mL^−1^) but different EC_50_ values against *B. neritina* (Compound **1**, 19.01 μmol mL^−1^ and Compound **2**, >185.19 μmol mL^−1^). It seems that the existence of the furan ring could affect the activity against *B. neritina*.

## 3. Experimental Section

### 3.1. Plant Material and Extraction

The fruits of *C. monnieri*, which were collected from Zhejiang Province, China in October 2006, were purchased from a local medicine store in Xiamen, Fujian Province, China. The plant material was identified by Dr. Yang Qiu, Department of Pharmacy, Xiamen University. A voucher specimen (SCZ-2006-10) is now deposited at the College of Oceanography and Environmental Science, Xiamen University. The fruits of *C. monnieri* (2.5 kg) were extracted with methanol (3 L) by maceration for 2 weeks at room temperature. The extract was desiccated by a vacuum rotary evaporator. The extract was dissolved in water and the solution was partitioned with EtOAc to obtain the EtOAc extract in yield of 35.6 g.

### 3.2. Isolation of Bioactive Compounds and Structural Identification

The EtOAc extract (35.6 g) from *C. monnieri* was chromatographed over a silica gel column using a gradient solvent system (petroleum ether-EtOAc = 20:1→1:1) to give five subfractions (F1–F5). The F2 fraction was separated over silica gel (petroleum ether-EtOAc = 12:1) to yield Compounds **1** (5 g) and **2** (750 mg). The F3 fraction was separated over silica gel (petroleum ether-EtOAc = 8:1) to yield Compound **3** (32 mg). The F4 fraction was separated over silica gel (petroleum ether-EtOAc = 5:1) and Sephadex LH-20 filtration (MeOH) to yield Compound **4** (12 mg).

The nuclear magnetic resonance (NMR) spectra were recorded with a Bruker Avance-600 FT NMR spectrometer (Bruker BioSpin GmbH, Rheinstetten, Germany) in CDCl_3_ with Tetramethylsilan (TMS) as a reference. electrospray ionization mass spectrometry (ESI-MS) data were recorded on an AB 3200Q TRAP spectrometer (AB SCIEX, Boston, MA, USA). Structural elucidation of the pure compounds was based on interpretation of their spectral data (NMR, MS) and comparison with published values.

### 3.3. Chemical Synthesis of Osthole Derivatives

Compounds **5**–**9** were prepared by standard organic synthesis procedures described in references [39,42,43], and the synthesis of these osthole derivatives is illustrated in Figure 2. Epoxide **5** was prepared by the epoxidation of **1** with *m*-chloroperbenzoic acid (*m*-CPBA). Diol **6** was synthesized from **5** by acid hydrolysis. Tertiary alcohol **7** was prepared by the oxymercuration of **1**. Aldehyde **8** was synthesized from **2** with the oxidant SeO_2_. Secondary alcohol **9** was prepared by the reduction of **8**. The synthetic procedures are described in the supplementary materials.

### 3.4. AF Assay

The barnacle *B. albicostatus* and the bryozoan *B. neritina* were used to test the AF activities of the natural products and synthetic derivatives. Adults of *B. albicostatus* were collected from the intertidal zone in Xiamen, Fujian Province, China. Based on the methods of references [9,20], after being released from the adults, the I–II stage nauplii were collected and reared to metamorphosis with *Chaetoceros muelleri* as food source. The larvae, which were metamorphosed to the cyprid stage, were stored in the dark at 5 °C until use for bioassays. Adult colonies of *B. neritina* were collected from a fish farm near Pozhao Island, Zhangzhou, Fujian Province, China. After exposure to the overhead room light, the adults released the larvae, which were harvested and immediately used [44]. Test samples were dissolved in EtOAc and the methods for measuring activities were based on references [9,20,44]. Percentages of larval settlement, swimming and death were calculated. The EC_50_ value (the concentration that reduced the settlement rate by 50% relative to the control) and LC_50_ value (the concentration that resulted in 50% mortality) of the compounds were calculated using the Spearman–Karber method [45]. The differences between the experimental treatments and controls were analyzed with one-way ANOVA followed by a Dunnet post hoc test. The significance level was defined as *p* < 0.05.

## 4. Conclusions

In conclusion, four coumarins were isolated from the herb *C. monnieri*, five other coumarins were prepared by chemical synthesis from osthol. All compounds were identified and tested for AF activities; most of them showed inhibitory activities against barnacle or bryozoan settlement. Among these compounds, osthole could be considered as a good lead compound in AF agent discovery, since it was present in a high quantity, was of simple structure and had substantial AF activities against both *B. albicostatus* and *B. neritina*. Furthermore, some preliminary information about the structure-activity relationship of these coumarins was given and the results showed that the groups on C-5′ and C-2′/C-3′ of the isoamylene chain could affect the AF activities.

## Figures and Tables

**Table 1:** Antilarval settlement activities of Compounds (**1**–**9**) against *Balanus albicostatus* and *Bugula neritina*.

Tested sample	*B. albicostatus*			*B. neritina*
	
	EC_50_(μg mL^−1^)	LC_50_(μg mL^−1^)	LC_50_/EC_50_	EC_50_(μg mL^−1^)
osthole (**1**)	4.64	37.42	8.06	7.56
imperatorin (**2**)	3.39	49.93	14.7	>50
isopimpinellin (**3**)	11.31	>50	UD	>50
auraptenol (**4**)	3.38	39.47	11.7	22.59
8-epoxypentylcoumarin (**5**)	7.46	>50	>6.7	37.29
Meranzin hydrate (**6**)	35.36	>100	UD	18.23
2′-deoxymetranzin hydrate (**7**)	4.67	>50	>10.7	9.06
8-methylbutenalcoumarin (**8**)	6.77	>100	>14.8	3.87
micromarin-F (**9**)	10.93	33.36	3.1	12.38
